# What does “urgency” mean when prioritizing cancer treatment? Results from a qualitative study with German oncologists and other experts during the COVID-19 pandemic

**DOI:** 10.1007/s00432-024-05863-7

**Published:** 2024-07-15

**Authors:** Sabine Sommerlatte, Helene Hense, Stephan Nadolny, Anna-Lena Kraeft, Celine Lugnier, Jochen Schmitt, Olaf Schoffer, Anke Reinacher-Schick, Jan Schildmann

**Affiliations:** 1 Institute for History and Ethics of Medicine, Interdisciplinary Center for Health Sciences, Medical Faculty, Martin Luther University Halle-Wittenberg, Magdeburger Str. 8, 06112 Halle (Saale), Germany; 2 Center for Evidence-Based Healthcare, Medical Faculty and University Hospital Carl Gustav Carus , TUD Dresden University of Technology, Dresden, Germany; 3 Institute for Educational and Health-Care Research in the Health Sector, Faculty of Health, Hochschule Bielefeld - University of Applied Sciences and Arts, Interaktion 1, 33619 Bielefeld, Germany; 4 Department of Hematology, Oncology and Palliative Care, St. Josef Hospital, Ruhr University Bochum, Bochum, Germany

**Keywords:** Empirical bioethics, Cancer care, Urgency, Resource allocation, Prioritization criteria, Pandemic

## Abstract

**Purpose:**

Cancer care in Germany during the COVID-19 pandemic was affected by resource scarcity and the necessity to prioritize medical measures. This study explores ethical criteria for prioritization and their application in cancer practices from the perspective of German oncologists and other experts.

**Methods:**

We conducted fourteen semi-structured interviews with German oncologists between February and July 2021 and fed findings of interviews and additional data on prioritizing cancer care into four structured group discussions, in January and February 2022, with 22 experts from medicine, nursing, law, ethics, health services research and health insurance. Interviews and group discussions were digitally recorded, transcribed verbatim and analyzed using qualitative content analysis.

**Results:**

Narratives of the participants focus on “urgency” as most acceptable criterion for prioritization in cancer care. Patients who are considered curable and those with a high level of suffering, were given a high degree of “urgency.” However, further analysis indicates that the “urgency” criterion needs to be further distinguished according to at least three different dimensions: “urgency” to (1) prevent imminent harm to life, (2) prevent future harm to life and (3) alleviate suffering. In addition, “urgency” is modulated by the “success,” which can be reached by means of an intervention, and the “likelihood” of reaching that success.

**Conclusion:**

Our analysis indicates that while “urgency” is a well-established criterion, its operationalization in the context of oncology is challenging. We argue that combined conceptual and clinical analyses are necessary for a sound application of the “urgency” criterion to prioritization in cancer care.

**Supplementary Information:**

The online version contains supplementary material available at 10.1007/s00432-024-05863-7.

## Background

The COVID-19 pandemic has fueled public and scientific attention on and debates about the equitable distribution of health resources (Emanuel et al. 2020; Marckmann et al. 2020; Trudeau et al. 2020; Wilkinson 2020). Although the debate focused initially on the care of COVID-19 patients and the distribution of intensive care resources, it quickly became evident that areas not primarily involved in the care of COVID-19 patients, such as cancer care, were also affected by limitations. Such limitations in oncology included a temporary reduction of cancer surgeries and a decline in screening and follow-up care (American Association for Cancer Research 2022; Eckford et al. 2022; Mazidimoradi et al. 2022, 2023; Oba et al. 2020; Reinacher-Schick et al. 2023; Rückher et al. 2022). The reservation of supply capacity for expected or actual corona patients (Lambertini et al. 2020; Leung et al. 2020; Reynolds et al. 2020; Stevens 2020) and the shortage of staff, due to, for example, infections with Sars-CoV-2 as well as other burdens (Lim et al. 2022; Schug et al. 2022; Sommerlatte et al. 2023; Tabur et al. 2022), were among the reasons for these changes.

In case of resource scarcity, any prioritization should be based on transparent and comprehensibly justified criteria and must take into account the supply reality of a country (Emanuel et al. 2020; ZEKO 2000). Accordingly, various national and international professional societies and other author groups have published recommendations for the prioritization of (cancer) care (American College of Surgeons 2020; Curigliano et al. 2020; Emanuel et al. 2020; Hanna et al. 2020; Marckmann et al. 2020; Marron et al. 2020; Meyfroidt et al. 2020; ÖGARI 2020; SAMW 2021). The well-established ethical criterion of “urgency” regarding allocation in case of scarcity was named as a principle to guide prioritization in many of these guidelines (American College of Surgeons 2020; Curigliano et al. 2020; Meyfroidt et al. 2020; SAMW 2021). However, while some recommendations mention “urgency” as an abstract criterion, other guidelines provide concrete priority lists without explaining how “urgency” was understood and translated into suggested rankings to prioritize certain diagnostic or therapeutic measures. This study aims to explore ethical criteria for prioritization and their application in cancer practices during the COVID-19 pandemic from the perspective of German oncologists and other experts.

Based on combined empirical-ethical analysis (see methods section), we focus on the operationalization of “urgency” in oncology and complementary criteria such as “success” and “likelihood of success”, which should guide allocation decisions. We argue that unlike in intensive care medicine, where “urgency” usually refers to the necessity to act in a timely manner in order to prevent imminent death and “likelihood of success” refers to the likelihood of surviving intensive care (Meyfroidt et al. 2020; Pugh et al. 2021; Marckmann et al. 2020; Bognar 2024), in oncology various therapeutic goals such as cure, prolongation of life and alleviation of suffering must be taken into account in the application of these criteria in prioritization decisions.

## Methods

The following presentation of the methodology and results follows the consolidated criteria for reporting qualitative research (COREQ) (Tong et al. 2007). The completed COREQ checklist is shown in Online Resource 1. Additional information concerning the credentials, occupation and gender of the researchers involved in this study is provided in Online Resource 2.

The data collection took place in two stages: (1) qualitative semi-structured interviews and (2) group discussions.

### Sampling

#### Qualitative interviews

We recruited a convenience sample of oncologists via the mailing list of members of the Working Group for Medical Oncology of the German Cancer Society (Arbeitsgemeinschaft Internistische Onkologie, *n* = 929). In addition, oncologists were contacted directly by the research team. All participants received written study information and informed consent form. Inclusion criteria were medical activity in a field of cancer medicine and consent to participate in the study. Exclusion criteria were lack of knowledge of the German language and lack of capacity to consent.

#### Group discussions

We applied the sampling strategy of a criterion-guided purposive selection in group discussions, using professional qualification as a criterion in order to capture the different perspectives of relevant stakeholders and experts and, thus, obtain as much variability of positions as possible (Akremi 2022). Purposive sampling achieves variance in participant characteristics and a heterogeneous sample (Schreier 2020).

The research team generated a list of relevant stakeholders from the fields of medicine, nursing, ethics, law, health services research, as well as health insurance and patient representatives. The experts received an invitation to participate in the group discussions via email and, if interested, written study information and an informed consent form. The inclusion criterion was proven expertise in the respective specialist area. Exclusion criteria were lack of knowledge of the German language and lack of capacity to consent.

### Data collection

#### Qualitative interviews

Based on a selective literature review and discussions within the research team, we developed an interview guideline on ethical challenges in dealing with scarce resources and prioritization criteria during the pandemic (Table 1). Fourteen qualitative semi-structured interviews were conducted via telephone (So, JS)^1^ between February and July 2021. The first two interviews were pilot interviews.

**Table 1:** Selected questions from the interview guide for physicians

	Questions
1	In which way has the pandemic affected the availability of certain resources, such as protective clothing or bed capacity in your area?
2	In some areas, the pandemic has led to deviations from standard patient care procedures, such as the postponement of diagnostic or therapeutic measures. Can you tell us what this was like for you?
3	What criteria were used to make prioritization decisions, e.g. who receives treatment and who is postponed?
4	Who was involved in the decision-making process?
5	How have decisions regarding such deviations been communicated with patients?
6	Was there anything that particularly worried you with regard to the best possible care for patients during the pandemic?
7	If you were the main person responsible for making prioritization decisions when resources are scarce, what would you pay particular attention to and what would be most likely to be dispensable?

#### Group discussions

We conducted four structured group discussions online on January 20, February 2, 7 and 15, 2022. They were each led by a moderator (JS) and supported by a co-moderator (HH, So)^2^. Two researchers took notes on important discussion points (HH, So) (Pohontsch et al. 2018). ARS^3^ was a participant observer in group discussion 1. The group discussions were structured according to three distinct topics: (1) diagnostics, screening and follow-up care, (2) tumor surgery and system therapy, and (3) psychosocial, and general and specialized palliative care.

Based on the analysis of quantitative and qualitative data collected beforehand by the CancerCOVID consortium (AIO and DGHO 2022), we created a PowerPoint presentation with key findings on the care of patients with colorectal and pancreatic cancer and verbatim quotes from the interviews (Table 2) to serve as stimuli for the group discussions. We focused on these two tumor entities because they represent the scientific focus of the interdisciplinary CancerCOVID research network, within the framework of which the group discussions were conducted (Lugnier et al. 2024). Due to their comparatively high incidence among both men and women, these entities are well suited as examples (Sung et al. 2021). Furthermore, we developed questions on criteria and possible justifications of these criteria for prioritizing (1) diagnostic procedures, (2) tumor surgery and systemic therapies, and (3) psychosocial and palliative care in the context of a current or future pandemic to stimulate discussions at the beginning of each topic (Table 2). Stimuli and questions were pilot tested within the research team and with other researchers at the Institute for History and Ethics of Medicine at Martin Luther University Halle-Wittenberg.

Both interviews and group discussions were digitally recorded as audio files and subsequently transcribed and anonymized during the process (Dresing et al. 2015).

**Table 2:** Questions and example of verbatim quote used as stimuli to facilitate group discussions on tumor surgery and system therapy

Questions
Imagine that in the context of the current or a future pandemic, there would be a reduction in available capacity for tumor surgery/system therapy.
1. In your view, what criteria should determine whether a patient with cancer is given priority or lower priority in terms of surgeries/system therapy?
2. How can you justify your prioritization?
**Example of verbatim quote**
[…] Someone who gets palliative therapy anyway is more likely to be postponed than someone who has a curative therapy approach. (INT 1, pos. 54)

### Data analysis

First, a preliminary analysis of the interviews was carried out in preparation for the group discussions. The second step involved analyzing the overall results of the interviews and group discussions. Analysis was based on the method of qualitative content analysis according to Kuckartz (2018). It includes the following 7 phases:

1. Initiatory work with the text, memo writing, case summaries.
2. Developing the main categories.
3. Coding the data with the main categories.
4. Inductive formation of subcategories.
5. Coding the data with the subcategories.
6. Simple and complex analyses (e.g. visualizations, tabular case summaries, relationships between the subcategories of a main category).
7. Writing up the results, documenting the procedure (Kuckartz 2018).

At the center of the method is the coding of the text using a category system. Thematically similar data sections were assigned so-called “codes” and summarized into superordinate main categories. First, the material was roughly coded using deductively generated codes, which were based on the questions in the interview guide and the topics of the group discussions. Further analysis was carried out using inductive coding of the material. Representative quotes were selected to illustrate the categories (printed in italics in the results section). Quotations were translated from German to English using DeepL software and checked by a native English speaker. Data coding and analysis of (sub)categories was performed using MAXQDA 2022. Additionally, we used pinboards to visualize the (sub)categories and relationships between them in order to refine the (sub)categories. Five researchers from the fields of medical ethics, medicine, nursing science, and health services research were involved in the analysis (So, HH, SN, JS, SD)^4^. The analysis of the group discussions focused on topic no. 2 (tumor surgery and system therapy), since difficult prioritization decisions and prioritization criteria were discussed in the interviews, particularly in relation to oncological therapies.

Empirical-ethical analysis was based on a consultative approach, according to which the study participants fed into the normative analysis by means of exploring their views and experiences (Davies et al. 2015). Examples for such an approach are “Reflexive Balancing” or ”Symbiotic Empirical Ethics” as proposed by Ives (2014) and Frith (2012). In line with core principles of this approach our methodology comprises the following overarching steps, which do run in an iterative process: setting out the circumstances and exploring morally relevant aspects of practice, specifying theories and principles, using ethical theory as a tool of analysis, theory building, and making normative judgements (Frith 2012).”

## Results

### Participants

We conducted and analyzed fourteen interviews, including two pilot interviews. Seven physicians were approached directly, 7 were recruited via mailing list. Each interview lasted between 12 and 54 min. The characteristics of the sample are shown in Table 3. The four group discussions each lasted between 72 and 76 min. Twenty-two experts from medicine, nursing, law, ethics, health services research and health insurance participated (Table 4).

**Table 3:** Selected characteristics of the physicians interviewed

Characteristics	Number of Participants
**Gender**	
Female	6
Male	8
**Qualification**	
Resident	7
Senior physician	4
Chief physician	3
**Specialty**	
Hematology/Oncology	12
Neuro-oncology	1
Urologic oncology	1
**Setting**	
Hospital	14
Medical office	1^a^

^a^This person worked both in a clinic and a medical office

**Table 4:** Selected characteristics of group discussion participants

	Number of Participants				
Characteristics	Group discussion 1	Group discussion 2	Group discussion 3	Group discussion 4	Total
Total N	5	6	7	4	22
**Gender**					
Male	3	4	6	4	17
Female	2	2	1	0	5
**Discipline**					
Ethics	0	0	1	1	2
Law	1	0	1	0	2
Medicine	4	4	3	2	13
Nursing	0	1	1	0	2
Health insurance	0	0	1	1	2
Health services research	0	1	0	0	1

### Findings

Main categories are shown in Table 5.^5^ Regarding (material) prioritization criteria in oncology, “urgency” and “(likelihood of) success” were at the center of both the interviews and the group discussions. Our analysis shows that both criteria have several dimensions when being considered in the context of cancer care. In the following, we present the identified dimensions first selected based on the narratives of the research participants and illustrate these dimensions using representative quotes as examples. We use pseudonyms for interviews (INT 1–14) and group discussions (GD 1–4) and identify the citations by means of abbreviations for the respective position (pos.) or page (p.).

**Table 5:** Main categories

Category	Description	Exemplary verbatim quote
1) Urgency	See page 10.	*In such a situation where resources are becoming scarce, the most obvious criterion for everyone is, I think, somehow urgency, right? You would first treat the person who particularly needs it now, because it’s essential for survival or because he has particularly severe symptoms or something like that. I think that would somehow make sense to everyone. (GD 1, p. 23, law expert)*
2) (Likelihood of) success	See page 11.	*[…] and that’s why it would be enough for me if you made a point of saying, well, where the prospects of success are good, we’ll give people a chance. (GD 4, p. 21, ethicist)*
3) Inadmissible criteria	Criteria that the experts largely considered inadmissible were religion and belief, comorbidity and disability as well as earning capacity.	*There are of course always criteria that don’t work. If you were to make distinctions according to discriminatory criteria, so to speak, which the Basic Law already prohibits, i.e. to name very extreme cases according to religion and ideology or something like that, that would not work, of course. (GD 1, p. 18, law expert)*
4) Procedural criteria	In addition to the material prioritization criteria, the importance of procedural criteria was also pointed out. With regards to procedural criteria, emphasis was placed on the need for transparency, i.e., prioritization decisions should be made according to established, visible criteria, and on consistency, i.e., the same criteria should be applied across patients and, ideally, across domains and institutions.	*And these formal criteria refer, for example, to the fact that it is carried out according to clearly defined criteria, that there is transparency, that there is formal equal treatment. (GD 3, p. 21, ethicist)*
5) Contextual factors	Some experts from the medical field reported that during the pandemic, criteria not directly related to the cancer or the health status of the respective patient sometimes also had an influence on prioritization decisions. Such contextual factors included, in particular, hygiene requirements during the pandemic.	*[…] because the performance of punctures etc. became much more complex with corona due to the need for a negative swab and so on and so forth. Then sometimes diagnostic things that I would normally say I could easily postpone for a week, I could NOT postpone because I wouldn’t have been able to get a new appointment and instead had to cancel people’s therapy appointments. (INT 4, pos. 47, physician)*

### Urgency

#### “Urgency” to prevent imminent harm to life

Some experts cite imminent harm to life, i.e. imminent death, as the greatest harm possible and a category on which the “urgency” of a therapeutic measure crucially depends. The patient with the smallest time window of opportunity to avert imminent death is considered most urgent.

*And as long as we get there with the criterion of urgency, I think there is a clear order of priority, i.e. the person who would die next is treated first in order to prevent them from dying, to avert the greatest harm. (GD 4, p. 24, ethicist)*

#### “Urgency” to prevent future harm to life

Furthermore, the threat of future harm to life, in the sense of a worsening of prognosis, was deemed relevant to determine the “urgency” of a therapeutic measure. Two types of future harm to life were discussed: a shortening of life expectancy in general, and particularly, that a disease which is deemed curable at the present stage will not be curable after postponement of a treatment.

*Then, for example, we have acute leukemia, which is a curable disease, even a well curable disease, but which urgently requires rapid initiation of treatment. We know very clearly that the prognosis will be worse if we wait unnecessarily long to start treatment. (INT 6, pos. 31, physician)*

*If surgery has already taken place, it is important to ensure that systemic therapy, if it is necessary, is given within a certain time frame, because we know, also from analyzing data, that if chemotherapy, e.g. for colon carcinoma, is postponed for longer than 3 weeks, survival is actually significantly worse. (GD 2, p. 20, expert from health services research)*

#### “Urgency” to alleviate suffering

The alleviation of suffering was another dimension of “urgency” discussed in the interviews and group discussions next to imminent or future harm to life. There is widespread agreement that patients with symptoms, such as severe pain, require urgent treatment due to the *high level of suffering* and deterioration of quality of life, even though the symptoms might not indicate an immediately life-threatening condition.

*[…] Particularly in oncology/palliative care, there may well be emergencies that require immediate treatment, intracranial pressure or something like that, and in order to maintain the quality of life as far as possible, very urgent pain therapy or something like that […]. (GD 1, p. 26, physician)*

### “(Likelihood of) success”

#### To heal or not to heal ‒ “(likelihood of) success” in the context of different treatment goals

In addition to the different dimensions of “urgency,” we found that the criterion of “urgency” was modulated by that of “success” and “likelihood of success.” In the following and based on the narratives in interviews and group discussions, “success” is understood as the achievement of a therapeutic goal and the actual realization of a potential benefit of a therapeutic measure. “Likelihood of success” in the strict sense, describes the probability of achieving a specific therapeutic goal, such as a cure, prolongation of life or alleviation of suffering.

A strong consideration was expressed in both the interviews and group discussions to prioritize curative patients over those for whom “only” a prolongation of life but no cure can be achieved.

*[…] so in the end mostly either curable patient versus non-curable patient, then you have to say that the decision was usually more in favor of the curable patient, or at least significantly more often. (INT 4, pos. 47, physician)*

*[…] I would like to add two points to the discussion, which are probably not entirely without controversy, namely that patients with a curative treatment approach naturally have a very high priority. And in particular the postponement of operations or multimodal therapy concepts for patients who can expect a curative approach is of course highly problematic. (GD 2, p. 20, physician)*

In this context, the criteria of “success” and “likelihood of success” were sometimes conflated. Consequently, no probabilities were compared (e.g. 80% chance of cure vs. 20% chance of cure), but, instead, a higher probability of “success” was attributed to patients who might achieve a higher benefit in terms of the years of life gained or with a curative therapy approach per se.

*So you would tend to take the one where you have the feeling that there is, how should I put it, a realistic, reasonable, perhaps even very good chance of cure? I mean, the difference is clear. It’s a different treatment goal, but this distinction between palliative and curative is a bit like saying that I’m going to take the person for whom I can actually do something curative and put the person I can only treat palliatively on the back burner. That goes a bit in that direction. I have no real prospect of success, so to speak. (GD 1, p. 23, law expert)*

## Discussion

This study explored ethical criteria for prioritization and their operationalization in cancer care during the COVID-19 pandemic from the perspective of oncologists and other experts. The criteria of “urgency” and “(likelihood of) success” were at the center of both the interviews and group discussions. Those are well-established criteria which are familiar from other medical fields, such as organ transplantation and intensive care medicine (Bobbert and Ganten 2013; Gottlieb 2017; Marckmann et al. 2020; ÖGARI 2020; SAMW 2021) and, at first sight, there seems to be a similarity to the allocation debate in critical care medicine. However, “urgency” in debates about allocating intensive care resources is – often implicitly – equated with urgency to avert harm, in the sense of loss of life, since intensive care units generally treat patients whose vital or organ functions are in a life-threatening condition and the aim is to save their lives and enable them to continue living as independently as possible outside the intensive care unit (Meyfroidt et al. 2020; Neitzke et al. 2019; Pugh et al. 2021). “Likelihood of success” is usually defined as the probability of surviving intensive care and getting discharged (Marckmann et al. 2020; Bognar 2024). Our analyses of interviews and group discussions revealed that the operationalization of these criteria in the context of oncology is much more challenging, because different dimensions of harm and, thus, diverse corresponding therapy goals, such as a cure, lifetime prolongation and the alleviation of suffering, must be taken into account (Markman 1994; Mieras et al. 2021). In addition, possible harm (e.g. loss of lifetime because a cancer is no longer curable due to delayed treatment) may lie in the future and, due to the probabilistic nature of future events and outcomes, might be less certain than in the case of rationing ventilators in intensive care, where death is imminent (Han et al. 2011).

Two groups of cancer patients, those for whom a cure can be achieved (1) and ones with a high level of suffering (2), were given high “urgency” (and, thereby, priority) by the interviewees. This observation might be explained by the fact that the interviewees implicitly refer to the rule of rescue, which states that the rescue of people must take place without question and as a priority, and emphasizes the importance of live-saving measures (McKie and Richardson 2003; Schöne-Seifert and Friedrich 2013). In this context, Schöne-Seifert and Friedrich (2013) distinguish between two types of “urgency.” In the first case, a therapeutic measure must be taken quickly in order to be successful at all (e.g. stopping an arterial hemorrhage). This would correspond in the case of our interviewees in the oncological context, for example, to the initiation of curative chemotherapy which may have to be carried out quickly (albeit with different time windows) so that a cure, i.e. a rescue, can still be achieved. In the second case, there is “urgency” because the existing condition (e.g. severe pain) is unbearable, even if it does not immediately lead to death or severe irreversible damage (Schöne-Seifert and Friedrich 2013). In oncology, for example, this could be the case for urgent pain therapy.

Furthermore, the consideration to prioritize “curative patients” is in line with some recommendations, such as those of the American Society of Clinical Oncology referring to the principle of maximizing health benefits, which can be operationalized as most lives saved (Emanuel and Persad 2023; Marron et al. 2020). While we agree that curing cancer is a great good, we think that giving unrestricted and unquestioned priority to patients with a curative treatment goal is a shortcut, which might entail the risk of systematically disadvantaging those patients who are not considered curable but for whom there is a high chance of gaining a significant extension of life.

Based on our findings from interviews and group discussions and further conceptual and ethical analyses, we suggest considering the following points when operationalizing “urgency,” “likelihood of success” and the “benefit of a therapy” in cancer care.

### Nuancing “urgency”

**“**Urgency” refers to the necessity to avert significant harm in a timely manner (Schöne-Seifert and Friedrich 2013). According to our data, different qualities of harm (immediate or future harm to life as well as immediate suffering) must be taken into account in oncology. “Urgency” is, therefore, a gradual and multidimensional criterion and must be assessed individually regarding harm and respective time windows for averting the damage (Schöne-Seifert and Friedrich 2013). The “urgent first” maxim is justifiable if it does not mean unreasonable sacrifices for those postponed (Schöne-Seifert and Friedrich 2013). We argue that, if a cancer can still be cured with a high likelihood in two weeks, it may, in certain cases, be ethically justifiable to postpone treatment within this time frame and to prioritize, for example, an urgent need to relieve existing suffering in a palliative situation.

### Distinguishing “success” from “likelihood of success”

It seems from some narratives that curative patients were given priority because the “likelihood of success” was conflated with “success” in the sense of the potential maximum benefit of a measure, i.e. curing the patient, while the likelihood to actually achieve that cure was hardly discussed. Our data suggest that it is necessary to clearly state what “curative” actually means in order to be able to make informed prioritization decisions in cancer care. We argue, that “curative,” from an ethical perspective, does not seem to be a decisional criterion if there is a patient for whom a cure is possible with a 20% chance, whereas for another “incurable” patient there might be a 90% probability that life could be significantly prolonged. Accordingly, it also seems to be important to identify those patients for whom a significant extension of life can still be achieved, rather than simply dividing patients into curable and non-curable. Doctors seem to be able to make a relatively accurate prognosis for patients who can still live for years (Orlovic et al. 2023).

### Nuancing “success”

Various dimensions of “success,” in the sense of actual benefit achieved, namely: cure, prolonging life and improving quality of life, are important in oncology (Markman 1994; Mieras et al. 2021). While it does not appear to us to be ethically unproblematic per se to simply prioritize curative treatment measures, it can make sense the other way round to identify measures that only have a small benefit or are “futile” (ZEKO 2022), as these would also be the measures whose postponement would cause the least harm and for which the Rule of Rescue is not deemed to be applicable (Schöne-Seifert and Friedrich 2013). Some authors suggest prioritization/rationing based on thresholds. A distinction is made between thresholds with low utility and those with a low chance of success (Schöne-Seifert et al. 2012). Withholding a potential benefit when there is little chance of success (e.g. cure in 20%) seems more problematic to Schöne-Seifert et al. (2012) than withholding a very small benefit even with a maximum response, i.e. a high chance of success, since in the former case an extremely high benefit may be expected in individual cases. The frameworks of the American Society of Clinical Oncology and the European Society for Medical Oncology concerning the benefit of diagnostic and treatment interventions and cancer drugs may provide a good evidence-based starting point regarding the respective decisions about prioritization (Cherny et al. 2015, 2017; Schildmann 2019; Schnipper et al. 2012).

### Limitations

This study focusses on the experiences of experts. The patient perspective has therefore not been analyzed. The interviews were mainly conducted with doctors from the clinical sector. Overall, only participants from Germany were included in the study. Transferability of our empirical findings to other contexts is, therefore, limited. Additionally, there may be social desirability bias since the prioritization of medical measures is ethically controversial and socially relevant, and some of the participating experts were representatives of professional interest groups. In addition, the ethical analysis focused on the prioritization of therapeutic measures. The prioritization of diagnostics, prevention and psychosocial and supportive palliative care and the significance of these areas of care were therefore not considered and should be addressed in future research.

## Conclusion

To the best of our knowledge, this is the first study that has explored and differentiated the well-established criterion of “urgency” in the context of cancer care based on the perspective of oncologists and other experts. According to German experts, “urgency” is acceptable for prioritizing therapeutic measures in oncology. However, the criterion must be operationalized in light of the different oncological treatment goals and regarding the maximum achievable “benefit” and “likelihood of success.” The results of this study have been incorporated into the development of an S1 guideline on prioritization in gastrointestinal tumors in the context of scarce resources (AIO and DGHO 2022; Lugnier et al. 2024).

## Electronic supplementary material

Below is the link to the electronic supplementary material.

Supplementary Material 1

Supplementary Material 2

## Data Availability

The data that support the findings of this study are available on request from the corresponding author. The data are not publicly available due to privacy or ethical restrictions.

## Footnotes

1 JS: Jan Schildmann; So: Sabine Sommerlatte.

2 HH: Helene Hense.

3 ARS: Anke Reinacher-Schick.

4 SD: Sophie Dahlke; SN: Stephan Nadolny.

5 As part of the iterative data analysis in the team, “urgency” and “likelihood of success” crystallized as central concepts that guided the group discussions. It also became evident, that the operationalization of these concepts in oncology differs significantly from other areas such as intensive care medicine. This does not seem to apply to the procedural and inadmissible criteria. We argue, that, for example, criteria such as transparency should apply to all areas of medicine in the same way. Prioritization according to religious belief should be equally inadmissible in both intensive care medicine and oncology. For this reason, the further analysis and presentation of the results focused on the categories of “urgency” and “likelihood of success”.
