# Supplementary material for: What does “urgency” mean when prioritizing cancer treatment? Results from a qualitative study with German oncologists and other experts during the COVID-19 pandemic
Source: J Cancer Res Clin Oncol. 2024 Jul 15;150(7):352. doi: 10.1007/s00432-024-05863-7 (PMC11249432; doi:10.1007/s00432-024-05863-7)
Supplement: Supplementary file 2 — Supplementary Material 2 [file 432_2024_5863_MOESM2_ESM.pdf]

Online Resource 2: Credentials, occupation and gender of the researchers at the time of the study

Article title: What does “urgency” mean when prioritizing cancer treatment? Results from a qualitative study with German oncologists and other experts during the COVID-19 pandemic

Journal: Journal of Cancer Research and Clinical Oncology

Authors: Sabine Sommerlatte, Helene Hense, Stephan Nadolny, Anna-Lena Kraeft, Celine Lugnier, Jochen Schmitt, Olaf Schoffer, Anke Reinacher-Schick, Jan Schildmann

Correspondence to:

Dr. med. Sabine Sommerlatte, M. mel.

Institute for History and Ethics of Medicine, Interdisciplinary Center for Health Sciences, Medical Faculty of Martin Luther University Halle-Wittenberg, Halle (Saale), Germany

sabine.sommerlatte@medizin.uni-halle.de

Credentials, occupation and gender of the researchers at the time of the study

Name	Credentials	Occupation	Gender
Sabine Sommerlatte	Physician, M. mel.*	Research associate	Female
Helene Hense	MSc	Research associate	Female
Stephan Nadolny	Dr. rer. medic.	Research associate	Male
Anna-Lena Kraeft	MD	Physician	Female
Celine Lugnier	MD	Physician	Female
Jochen Schmitt	MD, MPH	Director of the Center for Evidence-based Healthcare (ZEGV), University Hospital Carl Gustav Carus and Carl Gustav Carus Faculty of Medicine, Technische Universität Dresden	Male
Olaf Schoffer	Dr. rer. nat.	Research associate	Male
Anke Reinacher-Schick	MD	Director of the Department of Hematology, Oncology and Palliative Care, St. Josef Hospital, Ruhr University Bochum, Bochum	Female
Jan Schildmann	MD, MA	Director of the Institute for History and Ethics of Medicine, Martin Luther University Halle-Wittenberg	Male
Sophie Dahlke	Medical student	Student employee	Female

*Master of Medicine, Ethics and Law
